# Dynamic Coalition Formation among IoT Service Providers: A Systematic Exploration of IoT Dynamics Using an Agent-Based Model †

**DOI:** 10.3390/s24113471

**Published:** 2024-05-28

**Authors:** Joshua Shakya, Morgan Chopin, Leila Merghem-Boulahia

**Affiliations:** 1 Orange Innovation, 92320 Châtillon, France; morgan.chopin@orange.com; 2 LIST3N Laboratory, Université de Technologie de Troyes, 10300 Troyes, France; leila.merghem_boulahia@utt.fr

**Keywords:** internet of things, 5G networks, agent-based modeling, multi-agent systems, strategic collaborations, dynamic coalition formation

## Abstract

This paper introduces an Agent-Based Model (ABM) designed to investigate the dynamics of the Internet of Things (IoT) ecosystem, focusing on dynamic coalition formation among IoT Service Providers (SPs). Drawing on insights from our previous research in 5G network modeling, the ABM captures intricate interactions among devices, Mobile Network Operators (MNOs), SPs, and customers, offering a comprehensive framework for analyzing the IoT ecosystem’s complexities. In particular, to address the emerging challenge of dynamic coalition formation among SPs, we propose a distributed Multi-Agent Dynamic Coalition Formation (MA-DCF) algorithm aimed at enhancing service provision and fostering collaboration. This algorithm optimizes SP coalitions, dynamically adjusting to changing demands over time. Through extensive experimentation, we evaluate the algorithm’s performance, demonstrating its superiority in terms of both payoff and stability compared to three classical coalition formation algorithms: static coalition, non-overlapping coalition, and random coalition. This study significantly contributes to a deeper understanding of the IoT ecosystem’s dynamics and highlights the potential benefits of dynamic coalition formation among SPs, providing valuable insights and opening future avenues for exploration.

## 1. Introduction

The Internet of Things (IoT) [1,2,3] is a revolutionary paradigm that intertwines the physical and digital realms through interconnected devices. In this network, devices seamlessly communicate, generating vast streams of data that encapsulate the pulse of our interconnected world. The IoT ecosystem involves not only device interactions but also critical roles played by Mobile Network Operators (MNOs) and IoT Service Providers (SPs). MNOs, as the backbone of IoT networks, manage infrastructure for seamless communication among devices, ensuring reliability, scalability, and security. SPs contribute significantly by offering specialized applications, analytics, and value-added services built upon the foundation of IoT-generated data.

Despite the transformative potential and applications of IoT, the escalating complexity of its dynamics presents several challenges. Particularly, there is a noticeable gap in the existing literature concerning comprehensive studies that investigate the dynamics and interactions among IoT entities. While there are noteworthy IoT frameworks, such as the microservices-based architecture proposed in [4], the uAV IoT framework proposed in [5], and openIoT [6], these works often address specific aspects of IoT without delving into the granular and micro-level interactions among entities. The relationships among IoT entities can play a pivotal role in shaping the IoT ecosystem, giving rise to emergent behavior that could potentially provide insights into the enhancement of optimization strategies. However, current research efforts have yet to thoroughly explore this potential.

### 1.1. Research Objectives

To address this gap, we advocate for an Agent-Based Model (ABM) [7], a robust dynamic modeling approach that encapsulates the roles and interactions of entities. Agents, representing devices, MNOs, SPs, and more, authentically emulate real-world behaviors and collaborations, thereby enabling a nuanced exploration of IoT’s inherent complexity. This approach stands out from traditional methods by allowing for a meticulous examination of relationships and dynamics that might be overlooked in broader scopes. Furthermore, the adaptability and scalability of ABMs make them well suited for simulating the evolving nature of IoT ecosystems. New entities, behaviors, and environmental factors can be easily integrated into the model, allowing for the exploration of various scenarios and future developments in IoT networks. The potential of ABM for IoT extends beyond these aspects, and for a detailed examination of this potential, we refer the readers to [8].

As a use case within the ABM, we demonstrate the effectiveness of the model in simulating dynamic coalition formation among IoT SPs. Dynamic coalition formation, a critical phenomenon, involves SPs forming temporary alliances to achieve common goals. However, research on dynamic coalition formation among SPs in the IoT context is limited, with only a few studies in the literature, including [9,10]. While prior research, such as [9], has predominantly examined SP coalition formation through a pricing perspective and utilized bundling strategies, it often overlooks the complexities of large-scale collaboration scenarios, entailing numerous SPs engaging in multiple collaborations, competitions, and overlapping coalitions. This use case is particularly compelling in our context, as it effectively showcases the capabilities of the agent-based approach and the intricate dynamics discussed earlier, enhancing the practical relevance and applicability of the model.

### 1.2. Related Works

In the context of IoT, significant research efforts have been dedicated to exploring various aspects such as architectural considerations [11,12], device interactions [13], resource allocation, and optimization strategies [14]. Additionally, the utilization of ABM for IoT systems has gained recognition, with numerous papers [15,16] employing this approach to dissect and analyze IoT dynamics. Notably, ABM has been applied in diverse IoT use cases, ranging from smart waste monitoring [17] to self-adaptive and self-organizing applications [18].

Furthermore, our previous research work [19,20] delves into ABM within the realm of network slicing, introducing a Pricing Agent based on reinforcement learning (RL) to dynamically price network slices. This research lays a solid foundation for extending ABM methodologies to address the complexities of the IoT ecosystem. While our initial ABM focused on network slicing scenarios, its versatility suggests significant potential for IoT networks. In this paper, we extend the previous model by incorporating relevant agents specific to IoT and adjusting agent behaviors to suit IoT environments. In contrast to our previous work, which primarily investigated pricing mechanisms, this model delves into coalition formation dynamics within the IoT context.

While dynamic coalition formation has been extensively studied in various domains, ranging from applications in small cell networks [21] to smart grids [22], its exploration within IoT networks has garnered significant interest. Specifically, coalition formation mechanisms have been tailored for IoT device coalitions [23], aiming at optimizing energy and resource utilization in IoT environments. Many authors also approach coalition formation from a game theoretic perspective [24,25,26]. For example, in [24], an anti-jamming coalition formation game was proposed for Satellite-Enabled Army IoT, while [25] proposed machines that perform the task of IoT nodes forming clusters through a coalition formation game. Similarly, in [26], IoT operators formed coalitions to avoid intra-coalitional interference in terms of spectrum sensing. Despite this progress, the establishment of coalitions among SPs for effective service delivery remains a relatively underexplored area, with limited existing literature. Notably, a few studies have initiated investigations into this emerging topic, focusing on strategies such as bundling [9,10] and their implications for fostering collaborative arrangements among SPs. This evolving research direction holds promise for optimizing service delivery, enhancing resource utilization, and fostering collaborative innovation within the dynamic IoT ecosystem. Table 1 summarizes the comparison of different studies on dynamic coalition formation, positioning our work within the context.

### 1.3. Contributions

As is evident from the extensive discussion, the literature on IoT reveals numerous gaps, particularly with the absence of a proper modeling technique. Our work stands out as one of the few that advocates for an agent-based approach, which we believe is essential for capturing the complexities of IoT networks. Traditional modeling techniques often fail to adequately represent the dynamic and heterogeneous nature of IoT environments. In contrast, our proposed ABM offers a novel solution that allows for the simulation of individual entities with their own decision-making capabilities and interactions. This level of granularity enables a more realistic representation of the IoT ecosystem and facilitates the exploration of various use cases. Furthermore, our ABM is adaptable to a wide range of scenarios, making it a pioneering contribution to the field.

Furthermore, the emerging challenge of dynamic coalition formation among IoT SPs is an area that has received limited attention but is expected to be crucial in the future of IoT networks. To tackle this challenge, we propose a Multi-Agent Dynamic Coalition Formation (MA-DCF) approach. Unlike traditional coalition algorithms that rely on predefined structures, our novel approach leverages the adaptability of ABM to dynamically form and adjust coalitions based on real-time information and objectives. This innovative approach has demonstrated significant improvements in terms of efficiency and profitability compared to traditional methods, showcasing its potential to revolutionize collaborative strategies within the IoT ecosystem.

In summary, the key contributions of our work are as follows:

- Proposal of an adaptable and scalable ABM for the IoT Ecosystem: Firstly, our work introduces an ABM for the IoT ecosystem, which represents a departure from traditional modeling techniques. While existing methods often struggle to capture the dynamic and heterogeneous nature of IoT environments, our ABM allows for the simulation of individual entities, such as devices, MNOs, SPs, and customers, each with their own decision-making capabilities. This model ensures adaptability and scalability, effectively accommodating the dynamic nature of the IoT landscape. It serves as a practical and effective tool for researchers and practitioners seeking a systematic understanding of the multi-faceted interactions shaping the future of IoT.
- Demonstration of Model’s Versatility through a Practical Use Case: Another key contribution of the paper lies in its practical demonstration of the proposed ABM through a focused use-case scenario. Specifically, the paper explores the emerging challenge of dynamic formation of coalitions among IoT SPs—a critical aspect of the IoT ecosystem often overlooked in existing literature. Through this use-case scenario and its thorough experimentation, the paper not only showcases the versatility and effectiveness of the agent-based approach but also provides valuable insights into optimizing collaborative strategies and maximizing collective profits within the IoT ecosystem.

### 1.4. Paper Organization

The paper is organized as follows: Section 1 introduces the research problem, related works, main contributions, and provides an overview of the paper’s structure. Section 2 details our first contribution, the proposed Agent-Based Model (ABM) for IoT. Section 3 focuses on our second contribution, the Multi-Agent Dynamic Coalition Formation (MA-DCF) algorithm implemented on the IoT model. This section highlights the key elements of the algorithm and provides a detailed description of the simulation illustrating how the algorithm works, emphasizing its innovative approach to coalition formation among IoT SPs. Section 4 presents experimental results, including sensitivity analysis of key variables, performance assessment of the MA-DCF algorithm, and comparative analysis with other coalition formation algorithms. Finally, Section 5 concludes the paper, discussing implications and suggesting future research directions.

## 2. An Agent-Based Modelization for IoT

This section delves into the technical details of the ABM, purposefully designed to dissect the dynamics within the IoT ecosystem. Building upon prior work in modeling 5G networks with an agent-based approach [19,20], the model has been adapted to fit the specific characteristics of the IoT ecosystem. This evolution demonstrates the flexibility of the modeling framework to address diverse network architectures and underscores its adaptability to different technological paradigms. Functioning as a precision instrument, our proposed ABM IoT model comprises four key entities:

- Device agents ($A_{D}$): These agents represent the myriad IoT devices within the ecosystem, each equipped with specific functionalities and capabilities tailored to their intended purposes.
- Mobile Network Operator agents ($A_{M}$): Responsible for managing and orchestrating the communication infrastructure, these agents oversee the transmission of data between devices and SPs within the network.
- Service Provider agents ($A_{SP}$): These entities offer a range of services and solutions to customers with different needs and preferences by leveraging the data provided by device agents.
- Customer agents ($A_{C}$): Representing the end users and consumers of IoT services, these agents interact with SPs to access and utilize the offerings provided by the ecosystem.

Each entity is defined by a unique set of attributes that delineate their functionalities within the ecosystem. Interactions among these entities, governed by predefined rules, intricately shape the evolution of the IoT landscape. Additionally, the model incorporates dynamic coalition formation, introducing supplementary rules dictating the collaborative behaviors of SPs.

**Figure 1:**
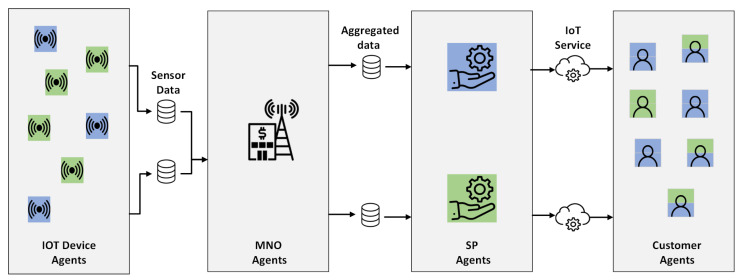
Schematic representation of the IoT ecosystem. IoT device agents and customer agents are color-coded to match their Service Provider (SP). Multiple colors for a customer indicate subscriptions to multiple SPs.

Figure 1 presents a detailed schematic of the IoT ecosystem, showcasing the interactions among its core components. At the center of the ecosystem, various IoT devices generate extensive data streams, which are efficiently aggregated and managed by the MNO agent. SP agents play a crucial role by strategically acquiring sensor data from the MNO and utilizing advanced processing techniques to offer a wide range of IoT services. Customers within the IoT ecosystem interact dynamically with the SPs, subscribing to the services that best align with their preferences and objectives. Importantly, customers may have varied needs and requirements, often necessitating the utilization of multiple services offered by different SPs. This dynamic demand for diverse services encourages collaboration and partnership among the SPs, leading to the formation of coalitions or alliances aimed at delivering comprehensive solutions to the users. While the figure illustrates data flow in one direction, it is important to note that there is significant interplay between the components in both directions. For instance, the preferences of customers can influence SP behavior and the selection of IoT devices, while feedback from SPs can shape the development and deployment of IoT devices. This reciprocal interaction contributes to the dynamic nature of the ecosystem and underscores the importance of considering multi-directional relationships. Overall, the figure provides a holistic representation of the IoT ecosystem, highlighting the interconnectedness of its key components and the collaborative dynamics that drive innovation and value creation within the ecosystem.

To provide an overview of the system dynamics, Algorithm 1 outlines the computational process. It commences with IoT device agents generating and transmitting data to their respective MNO agents. These MNO agents process and distribute the data to selected SP agents. Subsequently, customer agents select SPs based on their preferences and subscribe to them. The SP agents then execute the Multi-Agent Dynamic Coalition Formation (MA-DCF) algorithm (see Section 3) to evaluate their coalition status and offer both standalone and collaborative services. Finally, customers benefit from the services provided by the selected providers, thereby completing the iterative cycle. This orchestrated approach ensures efficient data flow, subscription processes, and service provision within the IoT landscape, catering to the diverse needs of both customers and SPs.
**Algorithm 1** High-level orchestrator algorithm for iot ecosystem dynamics.1:**while** true **do**2:      **for all** IoT Device Agents $A_{D_{i}}$ **do**3:            GenerateData($A_{D_{i}}$)4:            $A_{M_{i}}\leftarrow$ SelectMNO($A_{D_{i}}$)             ▹ Assigns the MNO associated with $A_{D_{i}}$5:            TransmitData($A_{D_{i}}$, $A_{M_{i}}$)6:      **end for**7:      **for all** Mobile Network Operator Agents $A_{M_{i}}$ **do**8:            ReceiveData($A_{M_{i}}$)9:            SelectedServiceProviders← SelectServiceProviders($A_{M_{i}}$) ▹ Selects the SPs contracted by $A_{M_{i}}$10:           **for all** SelectedServiceProviders$A_{SP_{k}}$ **do**11:                 TransmitData($A_{M_{i}}$, $A_{SP_{k}}$)12:           **end for**13:      **end for**14:      **for all** Customer Agents $A_{C_{j}}$ **do**15:            SelectedServiceProviders← SelectServiceProviders($A_{C_{j}}$)▹ Customer’s preferred SPs16:           **for all** $A_{SP_{k}}$ in SelectedServiceProviders **do**17:               Subscribe($A_{C_{j}}$, $A_{SP_{k}}$)18:          **end for**19:      **end for**20:      **for all** SP Agents $A_{SP_{i}}$ **do**21:          MA-DCF($A_{SP_{i}}$) ▹ See Section 322:          **if** $A_{SP_{i}}$ is part of a coalition **then**23:             OfferCollaborativeService($A_{SP_{i}}$)24:          **end if**25:          OfferStandaloneService($A_{SP_{i}}$)26:      **end for**27:      **for all** Customer Agents $A_{C_{j}}$ **do**28:          BenefitFromServices($A_{C_{j}}$)29:      **end for**30:**end while**

## 3. Simulation of a Practical Use Case: Multi Agent—Dynamic Coalition Formation (MA-DCF)

Coalitions in multi-agent systems, particularly within the IoT landscape, represent collaborative alliances formed by independent entities. These entities, often agents, pool their resources and expertise to collectively address challenges or capitalize on opportunities that may surpass individual capacities. Coalition formation involves strategic decision-making, where agents evaluate potential partners based on shared goals, complementary capabilities, and mutual benefits.

To unravel the intricacies of this dynamic coalition formation, we delve into the variables that govern the decision-making process and the evolving dynamics within this landscape:

- $A_{\mathrm{SP}}=\left\{A_{{\mathrm{SP}}_{0}},\ldots ,A_{{\mathrm{SP}}_{n}}\right\}$: Denotes the set of Service Provider agents in the system.
- $C=\{C_{0},\ldots ,C_{n}\}$: Represents all coalitions formed among Service Provider agents, where each coalition $C_{c}\subseteq A_{\mathrm{SP}}$.
- $Benefit\left(C_{c},t\right)\in \left[0,1\right]$: Represents the normalized benefit associated with coalition $C_{c}$ at time step *t*, indicating the overall advantage or gain obtained from the collaboration within that coalition.
- $Cost\left(C_{c},C_{c^{\prime }},t\right)\in \left[0,1\right]$: Denotes the normalized cost associated with coalition $C_{c}$ at time step *t*, including the joining cost and, if applicable, the leaving cost if the agent needs to exit coalition $C_{c^{\prime }}$ at time step *t*.
- $Utility\left(C_{c},C_{c^{\prime }},t\right)\in \left[-1,1\right]$: Represents the utility derived from coalition $C_{c}$ at time step *t*, calculated based on the difference between its benefit and the cost incurred to form the coalition, reflecting the overall satisfaction and effectiveness of collaboration within the coalition.

### 3.1. Core Matrices Guiding Multi-Agent Coalition Dynamics

In our simulation framework, we utilize four key matrices to capture various aspects of collaboration and compatibility among SPs within coalitions:

- Data Sharing Matrix (D): Represents the sharing of IoT devices between pairs of SPs at time step *t*, where $D_{ij}\left(t\right)\in \left[0,1\right]$ signifies the level of data sharing capability between SPs $A_{SP_{i}}$ and $A_{SP_{j}}$.
- Service Compatibility Matrix (S): Reflects co-subscription rates between pairs of SPs at time step *t*, with $S_{ij}\left(t\right)\in \left[0,1\right]$ incrementally increasing based on customer co-subscriptions, indicating a higher level of collaborative potential and compatibility between the involved providers.
- Resource Sharing Matrix (R): Represents the intersection of assigned tasks between SPs at time step *t*, with $R_{ij}\left(t\right)\in \left[0,1\right]$ values dynamically adjusted to signify high compatibility if two providers are engaged in identical computation tasks, indicating task redundancy.
- Joining Cost Matrix (X): Evaluates the cost of a SP joining a coalition with existing members at time step *t*, considering factors such as technological alignment and strategic fit. This matrix facilitates informed decision-making regarding coalition expansion or restructuring, where $X_{ij}\left(t\right)\in \left[0,1\right]$.

Matrices $D_{ij}\left(t\right)$, $S_{ij}\left(t\right)$, and $R_{ij}\left(t\right)$ play pivotal roles in shaping coalition dynamics among SPs $A_{SP_{i}}$ and $A_{SP_{j}}$ at time step *t*. These matrices, representing data sharing, service compatibility, and resource sharing, respectively, range from 0 to 1, denoting absence to high levels of capability or compatibility. As our simulation progresses, these matrices evolve dynamically, mirroring shifts in compatibility between SPs. For instance, $S_{ij}\left(t\right)$ responds to changing customer co-subscriptions, while $D_{ij}\left(t\right)$ reflects the utilization of shared IoT devices, reducing redundant data purchases. Meanwhile, $R_{ij}\left(t\right)$ identifies task redundancy, optimizing resource utilization. Finally, the joining cost matrix $X_{ij}\left(t\right)$ assesses the feasibility and cost of integrating members into a coalition based on technological alignment and strategic fit.

### 3.2. Benefit, Cost and Utility of Coalitions

Understanding the benefit, cost, and utility of coalitions is crucial for guiding strategic decision-making among SP agents. These metrics provide insights into the advantages and challenges associated with collaboration, ultimately influencing the formation and evolution of coalitions.

#### 3.2.1. Normalized Benefit of Coalition

In our analysis, the coalition benefit ($Benefit(C_{c},t)$) serves as a critical metric for assessing the overall advantage derived from collaboration within a specific coalition $C_{c}$ at time step *t*. The benefit is calculated using the formula:

(1) $$Benefit\left(C_{c},t\right)=\frac{2}{3|C_{c}\left|(\right|C_{c}\left|-1\right)}\left(\sum_{i,j\in C_{c}:i<j}D_{ij}\left(t\right)+\sum_{i,j\in C_{c}:i<j}S_{ij}\left(t\right)+\sum_{i,j\in C_{c}:i<j}R_{ij}\left(t\right)\right)$$

Here, $|C_{c}|$ denotes the number of agents within the coalition $C_{c}$, and $D_{ij}\left(t\right)$, $S_{ij}\left(t\right)$, and $R_{ij}\left(t\right)$ represent the values in the data sharing, service compatibility, and resource sharing matrices, respectively, between SPs $A_{SP_{i}}$ and $A_{SP_{j}}$ within the coalition at time step *t*. The term $\frac{2}{3|C_{c}\left|(|C|-1\right)}$ is utilized to normalize the benefit value between 0 and 1, ensuring standardized scaling across different coalition sizes and numbers of matrices. The factor of $\frac{1}{2}\left(\right|C_{c}\left|(\right|C_{c}\left|-1)\right)$ accounts for the total number of pairwise combinations within the coalition. Since there are three matrices (data sharing, service compatibility, and resource sharing) contributing to the benefit calculation, the factor of three is included to balance the influence of each matrix.

#### 3.2.2. Normalized Cost of Coalition

The normalized cost associated with a coalition at time step t, denoted as $Cost(C_{c},C_{c^{\prime }},t)$, comprises two components: the joining cost and the leaving cost. If a SP must exit an existing coalition before potentially joining another coalition, the leaving cost $Cost_{\mathrm{Leave}}\left(C_{c^{\prime }},t\right)$ is considered, where $C_{c^{\prime }}$ represents the coalition the SP intends to leave:

(2) $$Cost\left(C_{c},C_{c^{\prime }},t\right)=\frac{1}{2}\left(Cost_{\mathrm{Join}}\left(C_{c},t\right)+Cost_{\mathrm{Leave}}\left(C_{c^{\prime }},t\right)\right)$$

The join cost ($Cost_{\mathrm{Join}}\left(C_{c},t\right)$) represents the expense associated with a SP $A_{SP_{i}}$ joining a coalition $C_{c}$. The value in the joining cost matrix $X_{ij}\left(t\right)$ signifies the expense for SP $A_{{\mathrm{SP}}_{i}}$ to join a coalition $C_{c}$ containing member $A_{{\mathrm{SP}}_{j}}$ and time step *t*. By summing over all current coalition members $A_{{\mathrm{SP}}_{j}}$ within $C_{c}$ except $A_{{\mathrm{SP}}_{i}}$, the formulation accounts for the joining costs associated with each existing member. It is calculated as:

(3) $$Cost_{\mathrm{Join}}\left(C_{c},t\right)=\frac{1}{|C_{c}|-1}\sum_{j\in C_{c},j\neq i}X_{ij}\left(t\right)$$

If $C_{c^{\prime }}\neq \emptyset$, the leaving cost function involves two terms. The first term is denoted by f(δ(t)), where δ(t) represents the duration of the coalition. It is modeled as a sigmoid function that converges towards 1 as the duration of the coalition increases, capturing the increasing complexity and costliness of disengagement over time. Mathematically, the sigmoid function can be defined as:

(4) $$f\left(\delta \left(t\right)\right)=\frac{1}{1+e^{-\alpha (\delta (t)-\beta )}}$$

Here, α and β are parameters determining the shape of the sigmoid function.

Additionally, in the second term, $|C_{c^{\prime }}|$ denotes the number of SP agents within the coalition, and $|A_{\mathrm{SP}}|$ determines the total number of SP agents. As the coalition size grows, the leaving cost proportionally increases, reflecting the heightened intricacies and dependencies inherent in larger coalitions. Thus, the leaving cost dynamically adjusts based on both the temporal and structural dimensions of coalition dynamics.

The leaving cost function is mathematically represented as:

(5) $$Cost_{\mathrm{Leave}}\left(C_{c^{\prime }},t\right)=f\left(\delta \left(t\right)\right)\cdot \frac{|C_{c^{\prime }}|}{|A_{\mathrm{SP}}|}$$

It is important to note that if $C_{c^{\prime }}$ is empty, the leaving cost becomes 0.

#### 3.2.3. Utility of Coalition

The utility of a coalition at time step *t*, denoted as $Utility(C_{c},C_{c^{\prime }},t)$, signifies the net gain achieved by engaging in the coalition $C_{c}$ after accounting for associated costs, including potential impacts of departing from another coalition $C_{c^{\prime }}$. It is calculated as the difference between the benefit ($Benefit(C_{c},t)$) accrued from collaboration within $C_{c}$ and the total cost ($Cost(C_{c},C_{c^{\prime }},t)$) incurred to maintain membership in $C_{c}$, while also considering the potential cost of leaving $C_{c^{\prime }}$:

(6) $$Utility\left(C_{c},C_{c^{\prime }},t\right)=Benefit\left(C_{c},t\right)-Cost\left(C_{c},C_{c^{\prime }},t\right)$$

The utility of coalition $Utility(C_{c},C_{c^{\prime }},t)$ is constrained within the range $-1\leq Utility(C_{c},C_{c^{\prime }},t)\leq 1$, ensuring a normalized utility metric that offers a standardized measure of the net gain obtained from coalition participation. A positive utility value ($Utility(C_{c},C_{c^{\prime }},t)>0$) signifies a favorable inclination towards joining $C_{c}$, even after contemplating the potential cost of leaving $C_{c^{\prime }}$. Conversely, a zero or negative utility value ($Utility(C_{c},C_{c^{\prime }},t)\leq 0$) suggests that the incurred costs surpass the benefits, indicating a less desirable scenario for coalition participation.

### 3.3. Strategic Decision-Making

In the strategic decision-making process of SP agents, two critical elements drive their behavior: the commencement of coalition formation and the subsequent determination of whether to accept or decline coalition requests.

#### 3.3.1. Initiation of Coalition Formation

Each SP agent assesses its neighboring agents using various compatibility metrics, such as data sharing capabilities, service compatibility, and resource sharing potential, represented by matrices D, S, and R respectively. The compatibility between SP agents $A_{SP_{i}}$ and $A_{SP_{j}}$ at time step *t* is determined by the function:

(7) $$Compatibility\left(A_{SP_{i}},A_{SP_{j}},t\right)=\left\{\begin{array}{cc} 1, & \mathrm{if}\;D_{ij}\left(t\right)>\theta_{D}\;\mathrm{or}\;S_{ij}\left(t\right)>\theta_{S}\;\mathrm{or}\;R_{ij}\left(t\right)>\theta_{R}\\ 0, & \mathrm{otherwise} \end{array}\right.$$

where $\theta_{D}$, $\theta_{S}$, and $\theta_{R}$ are predefined thresholds. If the compatibility score between two SP agents exceeds any of the thresholds in the matrices, they are considered suitable partners for coalition formation.

To determine the compatible neighbors for a given SP agent $A_{SP_{i}}$ at time step *t*, the algorithm iterates through all neighboring agents $A_{SP_{j}}$ and evaluates their compatibility using the compatibility function. The set of compatible neighbors, denoted by $CompatibleNeighbors(A_{SP_{i}},t)$, is defined as:

(8) $$CompatibleNeighbors\left(A_{SP_{i}},t\right)=\left\{A_{SP_{j}}|Compatibility\left(A_{SP_{i}},A_{SP_{j}},t\right)=1\right\}$$

After assessing compatibility with neighboring agents, the SP agent forms a potential coalition by including itself and its compatible neighbors. If all members of this potential coalition accept the request, a new coalition is established. Mathematically, the decision to form a coalition, denoted by CoalitionFormation(PotentialCoalition,t), is determined by:

(9) $$CoalitionFormation\left(PotentialCoalition,t\right)=\left\{\begin{array}{cc} 1, & \mathrm{if}\;\forall A_{SP_{j}}\in PotentialCoalition,\\ \; & Acceptance(A_{SP_{j}},PotentialCoalition,t)=1\\ 0, & \mathrm{otherwise} \end{array}\right.$$

where $PotentialCoalition=\left\{A_{SP_{i}}\right\}\cup CompatibleNeighbors\left(A_{SP_{i}},t\right)$. The binary variable $Acceptance(A_{SP_{j}},PotentialCoalition,t)$ indicates whether SP agent $A_{SP_{j}}$ accepts the coalition request. This equation ensures that a new coalition is formed only if all potential members accept the coalition request.

#### 3.3.2. Acceptance or Rejection of Coalition Requests

When a SP $A_{{\mathrm{SP}}_{i}}$ receives a request for coalition formation, it can either accept or reject it. Let $Budget(A_{{\mathrm{SP}}_{i}})$ be the budget of $A_{{\mathrm{SP}}_{i}}$, representing the total number of coalitions it can join, and $Coalitions(A_{{\mathrm{SP}}_{i}},t)$ be the current coalitions of $A_{{\mathrm{SP}}_{i}}$. If the current number of coalitions $\left|Coalitions(A_{{\mathrm{SP}}_{i}},t)\right|$ is less than the budget, the SP accepts the request directly.

When the number of coalitions formed by SP $A_{{\mathrm{SP}}_{i}}$ reaches its budget $Budget(A_{{\mathrm{SP}}_{i}})$, the SP must carefully evaluate new coalition requests. In such cases, $A_{{\mathrm{SP}}_{i}}$ calculates the utility of the potential coalition by considering the cost of leaving one of its current coalitions $C_{c^{\prime }}$ at time *t*. Afterward, it selects the maximum utility among them and assesses whether this maximum utility is greater than 0. A utility greater than 0 signifies that leaving a coalition to join the new request would be beneficial.

The acceptance decision is determined by the following condition:

(10) $$Acceptance\left(A_{{\mathrm{SP}}_{i}},C_{c},t\right)=\left\{\begin{array}{cc} 1, & \mathrm{if}\;\left(|Coalitions\left(A_{{\mathrm{SP}}_{i}},t\right)|<Budget\left(A_{{\mathrm{SP}}_{i}}\right)\right)\;\mathrm{or}\\ \; & \;\left(|Coalitions\left(A_{{\mathrm{SP}}_{i}},t\right)|=Budget\left(A_{{\mathrm{SP}}_{i}}\right)\;\mathrm{and}\right.\\ \; & \;\left.\max_{C_{c^{\prime }}\in Coalitions\left(A_{{\mathrm{SP}}_{i}},t\right)}Utility\left(C_{c},C_{c^{\prime }},t\right)>0\right)\\ 0, & \mathrm{otherwise} \end{array}\right.$$

Here, $\max_{C_{c^{\prime }}\in Coalitions\left(A_{{\mathrm{SP}}_{i}},t\right)}Utility\left(C_{c},C_{c^{\prime }},t\right)$ represents the maximum utility obtained by evaluating the utility function for the coalition request $C_{c}$ with respect to each existing coalition $C_{c^{\prime }}$ at time *t*. If the maximum utility is greater than 0, the SP accepts the coalition request; otherwise, it rejects it.

### 3.4. Coalition and Individual Payoffs

Two critical metrics play significant roles in evaluating the effectiveness of our MA-DCF algorithm: coalition payoff and individual payoff. Coalition payoff reflects the collective benefit accrued from collaboration within formed coalitions, providing a measure of the overall success in achieving shared objectives and maximizing mutual gains. On the other hand, individual payoff assesses the net benefit obtained by individual SP agents participating in coalitions, capturing the effectiveness of their strategic decisions and the impact on their individual objectives and resources.

#### 3.4.1. Coalition Payoff

Coalition payoff represents the cumulative benefit derived from collaboration within a formed coalition at a specific time step *t*. Unlike the normalized benefit, coalition payoff does not involve normalization and provides a raw measure of the total advantage obtained from coalition participation. Mathematically, the coalition payoff ($Payoff(C_{c},t)$) at time step *t* is calculated as the sum of contributions from various factors such as data sharing ($D_{ij}$), service compatibility ($S_{ij}$), and resource sharing ($R_{ij}$) between all pairs of Service Provider (SP) agents $A_{SP_{i}}$ and $A_{SP_{j}}$ within the coalition $C_{c}$:

(11) $$Payoff\left(C_{c},t\right)=\left(\sum_{i,j\in C_{c}:i<j}D_{ij}\left(t\right)+\sum_{i,j\in C_{c}:i<j}S_{ij}\left(t\right)+\sum_{i,j\in C_{c}:i<j}R_{ij}\left(t\right)\right)$$

#### 3.4.2. Individual Payoff

The individual payoff refers to the net benefit received by a specific SP agent within a formed coalition, considering its contribution to the coalition’s overall payoff. It can be conceptualized as the difference between the coalition payoff with the individual SP ($Payoff(C_{c},t)$) and the coalition payoff without the individual SP ($Payoff(C_{c}\backslash \left\{A_{{\mathrm{SP}}_{i}}\right\},t)$). Mathematically, the individual payoff ($IndividualPayoff(A_{{\mathrm{SP}}_{i}},C_{c},t)$) for SP agent $A_{{\mathrm{SP}}_{i}}$ within coalition $C_{c}$ at time step *t* can be expressed as:

(12) $$IndividualPayoff\left(A_{{\mathrm{SP}}_{i}},C_{c},t\right)=Payoff\left(C_{c},t\right)-Payoff\left(C_{c}\backslash \left\{A_{{\mathrm{SP}}_{i}}\right\},t\right)$$

### 3.5. Description of Simulation

We now describe the MA-DCF, detailed in Algorithm 2, which simulates the process of SP agents forming coalitions over time by interacting with compatible neighbors and evaluating potential coalitions based on their individual utilities and constraints. To initialize the simulation, a population of agents is generated, and the environment is set up, including initializing the time step.
**Algorithm 2** Multi-agent dynamic coalition formation algorithm.1:Initialize simulation2:**while** t≤T **do**3:    t←t+14:    **for** each SP Agent $A_{SP_{i}}$ **do**5:        Get compatible neighbors: $CompatibleNeighbors(A_{SP_{i}},t)$6:        Get Potential Coalition: $PotentialCoalition\leftarrow \left\{A_{SP_{i}}\right\}\cup CompatibleNeighbors\left(A_{SP_{i}},t\right)$7:        **for** each $A_{SP_{j}}$ in PotentialCoalition **do**8:           **if** Budget($A_{SP_{j}}$) is reached **then**9:               Get $A_{SP_{j}}$ Coalitions at *t*: $Coalitions(A_{SP_{j}},t)$10:               **if** $\max_{ExistingCoalition\in Coalitions(A_{SP_{j}},t)}$ Utility(PotentialCoalition, ExistingCoalition, *t*) >0 **then**11:                   **if** $\mathrm{Acceptance}\left(A_{SP_{k}},PotentialCoalition,t\right)=1\;\forall A_{SP_{k}}\in PotentialCoalition$ **then**12:                       Leave Coalition ExistingCoalition13:                   **end if**14:               **end if**15:           **end if**16:        **end for**17:        **if** $\mathrm{Acceptance}\left(A_{SP_{j}},PotentialCoalition,t\right)=1\;\forall A_{SP_{j}}\in PotentialCoalition$ **then**18:           Enter Coalition PotentialCoalition19:        **end if**20:    **end for**21:**end while**22:End simulation

The simulation process unfolds in discrete time steps t=0,…,T, for $T\in N_{+}^{*}$. We will now describe the simulation step by step:

1. Initialize simulation:
  - The simulation is initialized by setting up the environment and generating a population of SP agents.
  - Time step *t* is initialized to 0.
2. Agent interaction and coalition formation:
  - For each SP agent $A_{SP_{i}}$ at each time step *t*, perform the following:
    - Obtain compatible neighbors using the function $CompatibleNeighbors(A_{SP_{i}},t)$.
    - Determine the potential coalition by including $A_{SP_{i}}$ and its compatible neighbors: $PotentialCoalition\leftarrow \left\{A_{SP_{i}}\right\}\cup CompatibleNeighbors\left(A_{SP_{i}},t\right)$.
    - Check if budget $Budget(A_{SP_{j}})$ is reached for each $A_{SP_{j}}$ in PotentialCoalition
      * If the budget is reached, and joining another coalition by leaving one of the current coalitions would increase utility, $A_{SP_{j}}$ waits for the potential coalition to be accepted by all parties. If it is accepted, the agent leaves one of the current coalitions, considering which one is most beneficial for its utility.
    - If the potential coalition is acceptable to all agents, they enter the coalition.
3. End of simulation:
  - The simulation ends after the time step *t* reaches *T*, where *T* is the predefined time limit.

**Figure 2:**
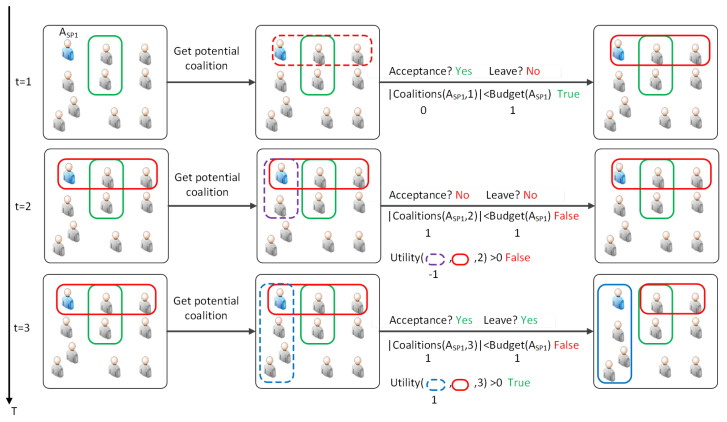
Evolution of coalition dynamics over time illustrating different strategies of SP agent $A_{SP_{1}}$ with $Budget(A_{SP_{1}})=1$. In the figure, dotted boxes represent potential coalitions and solid boxes represent formed coalitions. Each box is color-coded to represent a unique coalition.

To further enhance the understanding on coalition dynamics, Figure 2 illustrates the evolution of coalition dynamics over three time steps, focusing on the perspective of an SP, denoted as $A_{SP_{1}}$, with a budget of 1. As coalition formation requires acceptance from all members, it is assumed that all other members have already accepted the coalition, and we are awaiting the decision of $A_{SP_{1}}$. Each time step showcases a different dynamic of coalition formation.

- Time step t=1: Direct acceptance because budget constraint is not saturated. In the first time step, $A_{SP_{1}}$ forms a potential coalition with compatible neighbors. Since its budget is not saturated, $A_{SP_{1}}$ directly accepts the coalition offer without further evaluation. This scenario demonstrates a straightforward coalition formation, where the agent readily joins the coalition.
- Time step t=2: Rejection because of negative utility and budget constraint saturation. In the second time step, $A_{SP_{1}}$ has a potential coalition offer with one other member. However, $A_{SP_{1}}$ can only join one coalition due to its budget constraint. $A_{SP_{1}}$ rejects the new coalition offer because joining it would not result in a positive utility gain. Since the utility value of the new coalition offer and leaving the current one is less than zero, $A_{SP_{1}}$ rejects the offer, and no new coalition is formed. This scenario illustrates a cautious approach, where the agent considers the utility before accepting a new coalition.
- Time step t=3: Coalition change because of positive utility and budget constraint saturation. In the third time step, $A_{SP_{1}}$ receives another coalition proposal with three other members. After evaluating the utility, $A_{SP_{1}}$ determines that joining the new coalition and leaving the current one would yield a greater utility value. Consequently, $A_{SP_{1}}$ accepts the offer, leaves the previous coalition, and joins the new one. This scenario demonstrates the case where the agent maximizes its utility by switching coalitions.

## 4. Results

In the instantiation of our ABM for the IoT ecosystem, we meticulously define the parameters to reflect a realistic and dynamic simulation. The number of IoT Device Agents ($A_{D}$) is set at 1000, representing a diverse range of devices contributing to the IoT landscape. Mobile Network Operator Agents ($A_{M}$) are limited to 1, capturing the presence of a single operator managing the network infrastructure. SP agents ($A_{SP}$) are introduced with a count of 10, signifying a competitive yet collaborative environment with a variety of service offerings. Customer agents ($A_{C}$) are initialized to 500, representing the end users engaging with the IoT services. Each SP agent’s budget, reflecting the maximum number of coalitions it can join, ranges from one to five. This allocation, detailed in Table 2, signifies varying levels of strategic flexibility and collaboration potential within our simulated IoT ecosystem.

To initiate the simulation, we assume there are no existing coalitions among the SPs. Each SP operates independently, offering services separately to the IoT ecosystem. Therefore, the maximum number of coalitions (|C|) is initially set to 10, aligning with the number of SPs. This setup reflects the starting point, where SPs function individually without collaborative arrangements. Additionally, customers are subscribed to one or more SPs, with a higher probability of subscribing to a complementary SP, modeled using normal and mixed normal distribution.

The matrices representing capabilities and compatibilities between SPs within a coalition—D (data sharing), S (service compatibility), and R (resource sharing)—are initialized with random real numbers taken from the interval [0,1]. The matrices are given in Table 3. The initialization process is based on various factors, including the instantiation of SPs, and the customers’ initial subscription behavior. These matrices serve as the foundation for modeling the collaborative relationships between SPs within a coalition, and their values will evolve over time as the simulation progresses. Additionally, there is matrix X, which represents the cost for an SP to join a coalition considering all the members.

The simulation progresses through discrete time steps t=1,2,3,…,T, where *T* represents the total number of time steps defined. Each time step captures the actions and interactions among agents within the IoT ecosystem, including coalition formation, customer subscriptions, and resource exchange.

### 4.1. Sensitivity Analysis of Key Threshold Variables in MA-DCF

**Figure 3:**
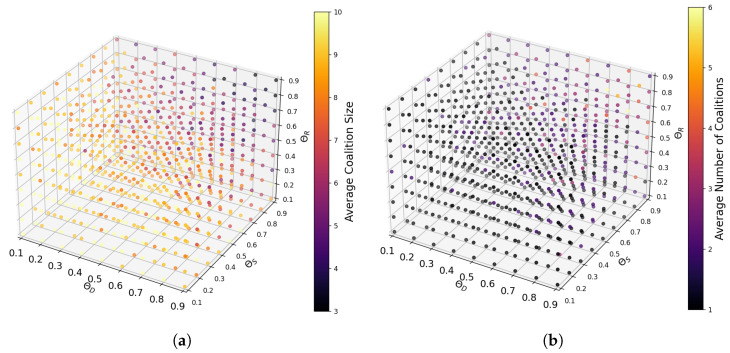
Impact of threshold values on coalition dynamics. (**a**) Variation in average coalition size with threshold combinations; (**b**) variation in average number of formed coalitions with threshold combinations.

The size and number of coalitions within the MA-DCF exhibit sensitivity to variations in threshold values $\theta_{D}$, $\theta_{S}$, and $\theta_{R}$. In our experimental setup, these thresholds are set within the range of 0.1 to 0.9 to elucidate their impact. Lower threshold values relax compatibility constraints, resulting in fewer but larger coalitions, as SP agents find it easier to meet the relaxed compatibility criteria. Conversely, higher threshold values impose stricter compatibility requirements, leading to the formation of many smaller and more exclusive coalitions. This nuanced relationship is illustrated in Figure 3a, showing how different combinations of threshold values correspond to varying average coalition sizes, and Figure 3b, which depicts the corresponding number of coalitions formed. Therefore, the precise calibration of threshold values is essential for shaping both the size and number of coalitions within the IoT ecosystem, ensuring that formed coalitions effectively leverage synergies and resources while maintaining compatibility among participating agents.

In our experimental setup, we opted for threshold values of 0.9 across all three metrics. This choice was motivated by the objective of striking a balance between fostering collaboration among SPs and maintaining compatibility standards. A higher threshold ensures that only highly compatible SPs are eligible for coalition formation, thereby promoting synergy and mutual benefit within the formed coalitions.

### 4.2. Performance Assessment of MA-DCF

**Figure 4:**
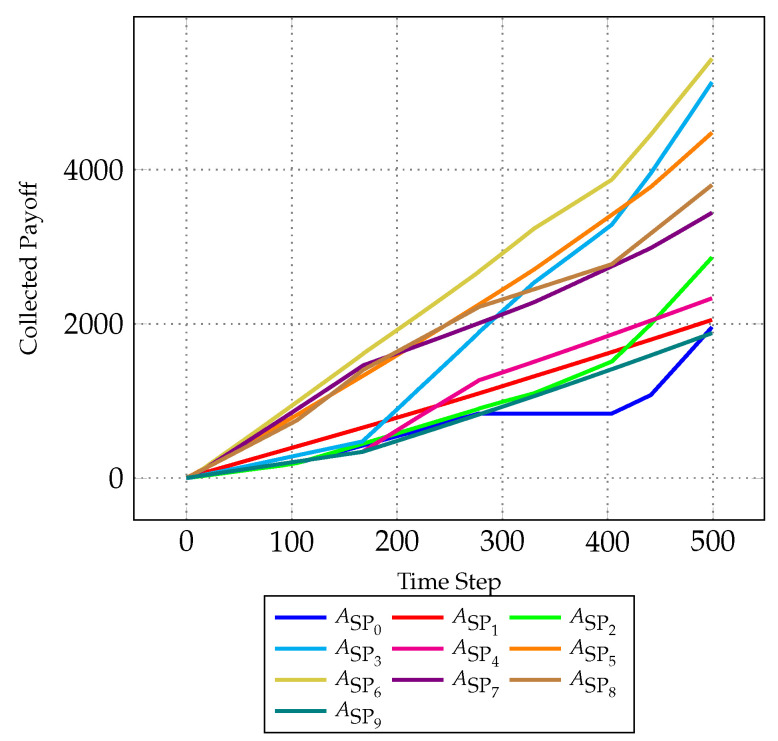
Evolution of collected individual payoff of each SP over time.

Figure 4 presents a comprehensive evaluation of the MA-DCF model’s performance, focusing on the aggregated collected payoff of individual SPs over successive time steps. The collected payoff of SP $A_{{\mathrm{SP}}_{i}}$ at time step *t* is obtained by summing the individual payoffs (refer to Equation (12)) for all coalitions $C_{c}$ in which $A_{{\mathrm{SP}}_{i}}$ is participating at each time step *t*:

(13) $$\sum_{C_{c}\in Coalitions\left(A_{{\mathrm{SP}}_{i}},t\right)}IndividualPayoff\left(A_{{\mathrm{SP}}_{i}},C_{c},t\right)$$

The payoff trajectory of each SP is depicted with a distinct line plot, enabling a granular analysis of their performance dynamics. The *x*-axis delineates time progression, offering a temporal context for assessing SP behavior, while the *y*-axis quantifies accumulated payoffs, reflecting the efficacy of coalition formations and strategic decisions.

A rigorous analysis of this figure is imperative for evaluating the efficacy of the MA-DCF model. For instance, sustained upward trends in the collected payoff of certain SPs, such as $A_{{SP}_{6}}$, may signify successful coalition formations or strategic alliances yielding favorable outcomes. Conversely, fluctuations or stagnation in payoff trajectories, like for SPs $A_{{SP}_{0}}$, may indicate challenges or inefficiencies in coalition dynamics, potentially necessitating further investigation into underlying factors such as negotiation strategies, or coalition stability. Comparative analysis across SPs facilitates the identification of performance disparities and strategic behaviors, informing refinements to the model and coalition formation strategies. This analysis can uncover the impact of factors such as SP budget allocations, resource availability, strategic decision-making on coalition dynamics, and overall performance.

**Figure 5:**
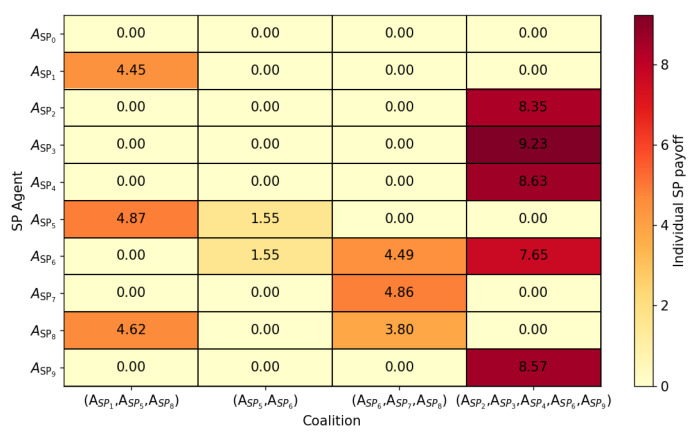
Heatmap illustrating individual SP payoffs from coalitions at the final simulation time step t = 500.

Figure 5 sheds light on the individual payoffs attained by each SP agent through participation in dynamic coalitions at a specific time step. By visually mapping the distribution of payoffs across different SP agents and coalition configurations, valuable insights can be gleaned regarding the efficacy of coalition formations. Additionally, it offers insights into the equitable distribution of payoffs among SP agents, facilitating the design of fair and sustainable coalition structures. Consider the analysis focused on the last coalition $\left(A_{{\mathrm{SP}}_{2}},A_{{\mathrm{SP}}_{3}},A_{{\mathrm{SP}}_{4}},A_{{\mathrm{SP}}_{6}},A_{{\mathrm{SP}}_{9}}\right)$. Here, SP agent $A_{{\mathrm{SP}}_{3}}$ emerges as the primary beneficiary, garnering the highest payoff due to its substantial contributions to the coalition’s objectives. Conversely, SP agent $A_{{\mathrm{SP}}_{6}}$ receives a notably lower payoff, underscoring variations in engagement levels and contributions among coalition members. It is worth noting that non-member SP agents receive a payoff of 0.0, indicative of their exclusion from coalition activities.

### 4.3. Comparative Analysis of MA-DCF: Performance Benchmarking

This analysis benchmarks the performance of the presented MA-DCF model against established baselines. By contrasting its strategic decision-making and compatibility matching with alternative scenarios, valuable insights are gained into its effectiveness and suitability for various applications.

- The static coalition baseline represents a fixed partnership approach, where coalitions remain unchanged throughout the process. While offering stability, it lacks the dynamic adaptations to resource availability and changing requirements inherent to the proposed model.
- The non-overlapping coalition baseline restricts SPs to single memberships, ensuring clear responsibilities and minimizing conflict. However, this approach might limit resource sharing and adaptability when tasks require diverse skillsets or resources scattered across multiple SPs.
- The random coalition baseline establishes a benchmark for improvement. Despite the potential for occasional compatibility through chance, this approach lacks strategic direction and is likely to cause the proposed model to underperform.

**Figure 6:**
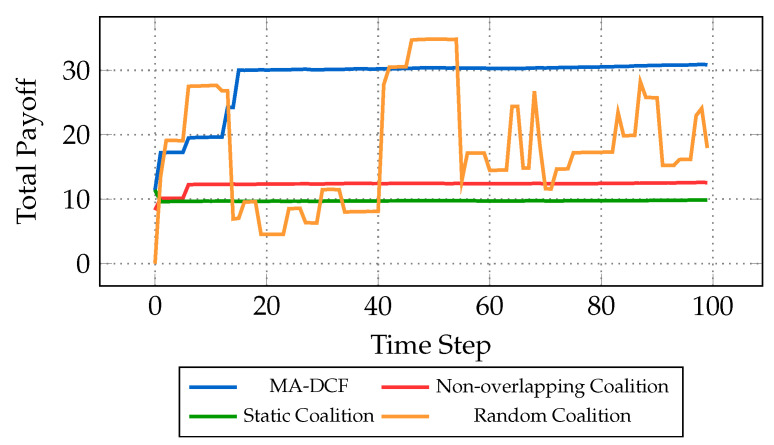
Temporal evolution of total payoff utilizing different coalition formation strategies.

Figure 6 serves as a visual depiction of the temporal evolution of total payoff using different coalition formation strategies. The total payoff at each time step, denoted as $\sum_{C_{c}\in C}\mathrm{Payoff}\left(C_{c},t\right)$, where *C* represents the coalitions formed using each approach, is analyzed across four distinct coalition formation strategies: MA-DCF, static coalition formation baseline, non-overlapping coalition formation baseline, and random coalition formation baseline. By iteratively optimizing coalition compositions based on agent preferences and environmental factors, MA-DCF ensures adaptability and responsiveness to evolving conditions. Conversely, the static coalition baseline predefines coalition structures without accounting for changing dynamics, potentially leading to suboptimal outcomes in volatile environments. The non-overlapping coalition baseline focuses on selecting the best coalition for each agent individually, aiming to maximize individual payoffs. However, this approach may inadvertently overlook opportunities for multiple collaborations among agents, thereby limiting the potential for synergistic partnerships and collective gain. By restricting agents to single coalitions, the non-overlapping coalition baseline may fail to exploit complementary skills and resources across different coalition configurations, leading to suboptimal overall performance. On the other hand, the random coalition baseline randomly selects coalitions, offering little strategic insight and resulting in unpredictable performance. This approach, while occasionally yielding favorable outcomes, lacks strategic foresight and stability, rendering its performance unpredictable and unsuitable for robust decision-making processes.

**Figure 7:**
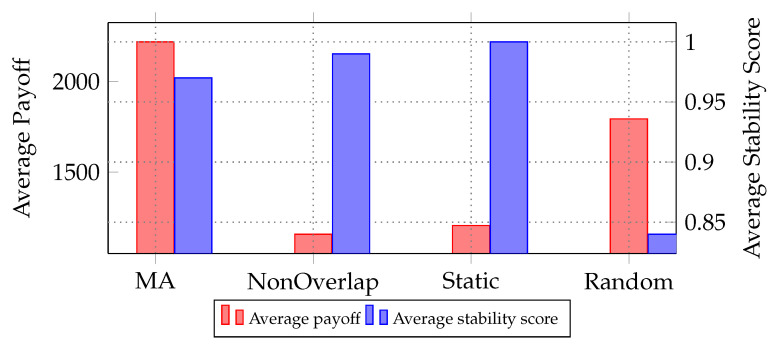
Comparison of average payoff and stability score for each approach over 10 iterations, each comprising 100 steps.

Figure 7 further delves into the comparison between the four coalition formation strategies, providing a deeper analysis of their performance and stability. This detailed analysis is derived from a comprehensive simulation comprising 10 runs, each spanning 100 time steps. The average payoff, depicted on the left *y*-axis, represents the payoff (refer to Equation (11)) amassed by coalitions formed using each approach after 100 time steps, averaged across the 10 runs. It can be calculated as:

(14) $$\frac{1}{N}\sum_{i=1}^{N}\sum_{t=1}^{T}\sum_{C_{c}\in C}Payoff\left(C_{c},t\right)$$

where *N* is the number of runs, *T* is the number of time steps, and *C* represents the set of coalitions formed using each approach at time step *t*.

On the right *y*-axis, the stability metric is introduced to evaluate the consistency and resilience of coalition formations over time. This metric, calculated as the average stability using Jaccard’s index [27] between two consecutive time steps in each run, provides deeper insights into the dynamics of coalition structures. It can be computed as:

(15) $$\frac{1}{N}\sum_{i=1}^{N}\frac{1}{T-1}\sum_{t=2}^{T}J\left(C_{i,t-1},C_{i,t}\right)$$

where $J(C_{i,t-1},C_{i,t})$ denotes the Jaccard’s index between coalition structures at time steps t−1 and *t* in the current iteration.

Notably, the MA-DCF approach emerges as the most promising, yielding the highest average payoff after 100 time steps. This underscores its efficacy in orchestrating dynamic coalitions to maximize collective gains. Conversely, the non-overlap baseline exhibits the lowest average payoff, reflecting its limited capability to foster collaboration among agents. Meanwhile, both the random and static coalition baselines present mixed outcomes, with their respective payoffs fluctuating inconsistently. Delving into stability, the static approach demonstrates remarkable stability, maintaining a consistent coalition structure throughout the simulation. In contrast, the random baseline displays the lowest stability, characterized by frequent coalition reconfigurations that could potentially incur additional costs. Notably, the MA-DCF approach, along with the Static baseline, demonstrates respectable stability, suggesting their reliability in dynamic environments. Taking into consideration all factors, the MA-DCF approach emerges as the superior choice in terms of both profitability and stability compared to the alternatives provided.

## 5. Conclusions

In summary, our Agent-Based Model (ABM) serves as a powerful tool for dissecting the intricate dynamics within the Internet of Things (IoT) ecosystem. By integrating dynamic coalition formation among Service Providers (SPs), our Multi-Agent Dynamic Coalition Formation (MA-DCF) algorithm integrated in the ABM adeptly captures the adaptive nature inherent in collaborations amidst the evolving IoT landscape. This adaptability empowers SPs to strategically navigate coalition formations based on mutual benefits, offering invaluable insights into the complex interplay of collaborative strategies within the IoT domain. We conduct a sensitivity analysis of key threshold variables in the MA-DCF model, demonstrating how variations in threshold values impact the size and number of coalitions formed, emphasizing the importance of the precise calibration of threshold values to shape coalition dynamics effectively. Furthermore, we assess the performance of the MA-DCF model by evaluating the collected payoff of individual SPs over successive time steps. We analyze payoff trajectories to identify successful coalition formations and strategic behaviors, facilitating improvements to the model and coalition formation strategies. Notably, our research has yielded compelling results, demonstrating the efficiency of our MA-DCF algorithm over three baseline coalition formation algorithms in terms of both payoff and stability. This empirical validation not only reinforces the efficacy of our approach in optimizing outcomes within the IoT ecosystem but also solidifies the model’s foundational role in comprehensively assessing both present and future states of IoT interactions.

Looking ahead, our study underscores several key technical avenues for further exploration. Firstly, a deeper dive into subscriber behavior analysis is essential to unravel the impact of coalition formations on consumer preferences, adoption patterns, and loyalty dynamics across diverse coalition structures. This involves employing advanced data analytics techniques and behavioral modeling methodologies to glean insights into the complex interplay between coalition strategies and subscriber behavior within the IoT ecosystem. Additionally, a more granular exploration of market dynamics modeling holds immense potential for uncovering the interplay of factors such as pricing strategies, competitive dynamics, and the influx of new market entrants, all of which are significantly influenced by coalition formations over time. Complementing these avenues, an examination of the effects of coalitions on service quality metrics—ranging from network reliability and data security to customer support—will be pivotal in ensuring the longevity and trustworthiness of IoT services. By directing our research efforts along these technical trajectories, we can deepen our understanding of coalition dynamics within the IoT ecosystem and develop informed strategies that drive the field towards greater resilience and innovation.

## Figures and Tables

**Table 1:** Comparison of different studies on dynamic coalition formation.

Study	Domain	Approach	Key Findings
[21]	Small cell networks	Game theory	Proposed a game theoretic approach for dynamic coalition formation to cluster the small cell base stations so that they can perform cluster-wise joint beamforming.
[22]	Smart grids	Game theory	Investigated dynamic coalition formation in smart grids, aiming at efficient energy distribution and management.
[23]	IoT device coalitions	Optimization	Proposed coalition formation and resource management among Machine-to-Machine communication devices in IoT applications, considering factors such as energy availability, communication interests, and physical ties.
[24]	Satellite-Enabled Army IoT	Game theory	Proposed a distributed dynamic anti-jamming scheme for Satellite-enabled Army Internet of Things to decrease energy consumption in jamming environments, utilizing hierarchical anti-jamming Stackelberg and coalition formation games, along with reinforcement-learning-based algorithms.
[25]	IoT nodes clustering	Game theory	Proposed a smart city architecture with dew computing and machine-to-machine communication for IoT, where machines form clusters through coalition formation games to optimize task distribution, resulting in significantly lower delay and power consumption compared to non-optimized networks.
[26]	IoT operators	Game theory	Investigated an opportunistic Cognitive Radio Network where IoT operators form coalitions to access licensed spectrum, utilizing a Sensing Agent to improve data rate and reduce energy consumption, and employing Nash bargaining to ensure economic fairness and throughput improvement.
[9]	IoT SPs	Bundling strategy	Proposed a smart data pricing approach for managing IoT data efficiently, determining sensing data buying price, IoT service subscription fee, and bundling strategy to attract more users and achieve higher revenue.
[10]	IoT SPs	Optimization	Introduced optimal pricing and bundling schemes for machine learning-based IoT services, maximizing profit through standalone and bundled sales while employing cooperative game theory for fair profit sharing among Service Providers.
**Our Work**	**IoT SPs**	**Multi-agent approach**	**Proposes dynamic coalition formation among IoT SPs using a multi-agent approach, considering realistic interactions and behaviors. Demonstrates the potential to optimize service delivery, payoffs, and foster collaborative innovation.**

This table provides a comparative overview of different domains, approaches, and key findings in the field of dynamic coalition formation. Our study is highlighted to contextualize its contributions relative to other research in this area.

**Table 2:** Budget allocation for Service Providers.

Service Provider $\left(A_{{\mathrm{SP}}_{i}}\right)$	$A_{{\mathrm{SP}}_{0}}$	$A_{{\mathrm{SP}}_{1}}$	$A_{{\mathrm{SP}}_{2}}$	$A_{{\mathrm{SP}}_{3}}$	$A_{{\mathrm{SP}}_{4}}$	$A_{{\mathrm{SP}}_{5}}$	$A_{{\mathrm{SP}}_{6}}$	$A_{{\mathrm{SP}}_{7}}$	$A_{{\mathrm{SP}}_{8}}$	$A_{{\mathrm{SP}}_{9}}$
$Budget(A_{SP_{i}})$	2	3	2	4	4	2	4	5	4	3

**Table 3:** Initial state of matrices: data sharing (D), service compatibility (S), resource sharing (R), and joining cost (X) at simulation time step t = 0.

$D=\left[\begin{array}{cccccccccc} 0.0 & 0.5 & 0.9 & 0.1 & 0.6 & 0.4 & 0.8 & 0.4 & 0.2 & 0.3\\ 0.5 & 0.0 & 0.7 & 0.8 & 0.5 & 0.4 & 0.4 & 0.6 & 0.3 & 0.1\\ 0.9 & 0.7 & 0.0 & 0.1 & 0.3 & 0.2 & 0.7 & 0.6 & 0.1 & 0.3\\ 0.1 & 0.8 & 0.1 & 0.0 & 0.7 & 0.0 & 0.9 & 0.1 & 0.5 & 0.7\\ 0.6 & 0.5 & 0.3 & 0.7 & 0.0 & 0.3 & 0.6 & 0.2 & 0.8 & 0.4\\ 0.4 & 0.4 & 0.2 & 0.0 & 0.3 & 0.0 & 0.1 & 0.4 & 0.4 & 0.5\\ 0.8 & 0.4 & 0.7 & 0.9 & 0.6 & 0.1 & 0.0 & 0.9 & 0.2 & 0.1\\ 0.4 & 0.6 & 0.6 & 0.1 & 0.2 & 0.4 & 0.9 & 0.0 & 0.7 & 0.9\\ 0.2 & 0.3 & 0.1 & 0.5 & 0.8 & 0.4 & 0.2 & 0.7 & 0.0 & 0.8\\ 0.3 & 0.1 & 0.3 & 0.7 & 0.4 & 0.5 & 0.1 & 0.9 & 0.8 & 0.0 \end{array}\right]$	$S=\left[\begin{array}{cccccccccc} 0.0 & 0.1 & 0.6 & 0.8 & 0.9 & 0.7 & 0.2 & 0.4 & 0.8 & 0.7\\ 0.1 & 0.0 & 0.1 & 0.1 & 0.9 & 0.9 & 0.4 & 0.5 & 1.0 & 0.9\\ 0.6 & 0.1 & 0.0 & 0.9 & 0.2 & 0.3 & 0.8 & 0.1 & 0.6 & 0.8\\ 0.8 & 0.1 & 0.9 & 0.0 & 0.4 & 0.0 & 0.5 & 0.2 & 0.2 & 0.9\\ 0.9 & 0.9 & 0.2 & 0.4 & 0.0 & 0.3 & 0.0 & 0.2 & 0.2 & 1.0\\ 0.7 & 0.9 & 0.3 & 0.0 & 0.3 & 0.0 & 1.0 & 0.3 & 1.0 & 0.2\\ 0.2 & 0.4 & 0.8 & 0.5 & 0.0 & 1.0 & 0.0 & 0.9 & 0.4 & 0.6\\ 0.4 & 0.5 & 0.1 & 0.2 & 0.2 & 0.3 & 0.9 & 0.0 & 0.1 & 0.4\\ 0.8 & 1.0 & 0.6 & 0.2 & 0.2 & 1.0 & 0.4 & 0.1 & 0.0 & 0.1\\ 0.7 & 0.9 & 0.8 & 0.9 & 1.0 & 0.2 & 0.6 & 0.4 & 0.1 & 0.0 \end{array}\right]$
$R=\left[\begin{array}{cccccccccc} 0.0 & 0.3 & 0.3 & 0.5 & 0.2 & 0.7 & 0.0 & 0.4 & 0.2 & 0.4\\ 0.3 & 0.0 & 0.2 & 0.5 & 0.8 & 1.0 & 0.8 & 0.6 & 0.3 & 0.8\\ 0.3 & 0.2 & 0.0 & 0.8 & 0.6 & 0.1 & 0.2 & 0.0 & 0.8 & 0.3\\ 0.5 & 0.5 & 0.8 & 0.0 & 0.7 & 0.1 & 0.7 & 0.2 & 0.5 & 0.6\\ 0.2 & 0.8 & 0.6 & 0.7 & 0.0 & 0.6 & 0.5 & 0.8 & 0.3 & 0.6\\ 0.7 & 1.0 & 0.1 & 0.1 & 0.6 & 0.0 & 0.2 & 0.9 & 1.0 & 0.7\\ 0.0 & 0.8 & 0.2 & 0.7 & 0.5 & 0.2 & 0.0 & 0.6 & 0.7 & 0.3\\ 0.4 & 0.6 & 0.0 & 0.2 & 0.8 & 0.9 & 0.6 & 0.0 & 1.0 & 0.5\\ 0.2 & 0.3 & 0.8 & 0.5 & 0.3 & 1.0 & 0.7 & 1.0 & 0.0 & 0.8\\ 0.4 & 0.8 & 0.3 & 0.6 & 0.6 & 0.7 & 0.3 & 0.5 & 0.8 & 0.0 \end{array}\right]$	$X=\left[\begin{array}{cccccccccc} 0.0 & 0.1 & 0.8 & 0.9 & 0.4 & 0.3 & 0.5 & 0.4 & 0.1 & 0.1\\ 0.1 & 0.0 & 0.0 & 0.5 & 0.9 & 0.3 & 0.6 & 0.0 & 0.4 & 0.1\\ 0.8 & 0.0 & 0.0 & 0.9 & 0.7 & 0.1 & 0.1 & 0.4 & 0.6 & 0.4\\ 0.9 & 0.5 & 0.9 & 0.0 & 0.0 & 0.6 & 0.2 & 0.3 & 0.6 & 0.7\\ 0.4 & 0.9 & 0.7 & 0.0 & 0.0 & 1.0 & 1.0 & 0.2 & 0.3 & 0.5\\ 0.3 & 0.3 & 0.1 & 0.6 & 1.0 & 0.0 & 0.5 & 0.0 & 0.4 & 0.6\\ 0.5 & 0.6 & 0.1 & 0.2 & 1.0 & 0.5 & 0.0 & 1.0 & 0.1 & 0.1\\ 0.4 & 0.0 & 0.4 & 0.3 & 0.2 & 0.0 & 1.0 & 0.0 & 0.9 & 0.4\\ 0.1 & 0.4 & 0.6 & 0.6 & 0.3 & 0.4 & 0.1 & 0.9 & 0.0 & 0.1\\ 0.1 & 0.1 & 0.4 & 0.7 & 0.5 & 0.6 & 0.1 & 0.4 & 0.1 & 0.0 \end{array}\right]$

## Data Availability

The data presented in this study are available on request from the corresponding author.
